# Effects of organic and inorganic selenium mixes in pregnant sows on piglet growth, selenium levels in serum and milk, and selenium deposition in newborn piglet tissues

**DOI:** 10.5713/ab.24.0661

**Published:** 2024-10-07

**Authors:** Xing Hao Jin, Min Soo Park, Min Hyuk Jang, Cheon Soo Kim, Yoo Yong Kim

**Affiliations:** 1 Department of Agricultural Biotechnology and Research Institute of Agriculture and Life Science, Seoul National University, Seoul 08826, Korea

**Keywords:** Gestating Sows, Litter Performance, Selenium Source, Selenium Status

## Abstract

**Objective:**

This study was conducted to evaluate the effects of organic and inorganic selenium mixes in pregnant sows on piglet growth, selenium levels in serum and milk, and selenium deposition in newborn piglet tissues.

**Methods:**

A total of 44 multiparous sows (Yorkshire×Landrace) with average body weight (BW), backfat thickness, and parity were assigned to one of the three treatments with 14 or 15 sows per treatment in a completely randomized design. The treatments were as follows: i) Control, corn-soybean meal-based diet with no addition of selenium premix; ii) ISOS (mixed inorganic selenium and organic selenium) 30, a basal diet supplemented with 0.15 ppm of inorganic Se and 0.15 ppm of organic Se; iii) ISOS50, a basal diet supplemented with 0.25 ppm of inorganic Se and 0.25 ppm of organic Se.

**Results:**

At day 21 of lactation, supplementing a high level of mixed Se at 0.50 ppm resulted in higher piglet BW and weight gain than adding a low level of mixed Se at 0.30 ppm (p<0.05). Selenium concentration of colostrum in sows fed ISOS50 diet was significantly higher than those in sows fed ISOS30 diet (p<0.05). Selenium concentrations in the serum at days 90 and 110 of gestation and 24 hours postpartum were highest when sows were fed ISOS50 diet (p<0.05). Additionally, increasing levels of mixed Se led to an increase in piglet serum Se concentration at 24 hours postpartum (p<0.05). Before ingesting colostrum, piglets from sows fed a mixed selenium (Se) diet had significantly higher kidney Se concentrations compared to those from the control group, with the ISOS50 treatment showing the most significant difference (p<0.05).

**Conclusion:**

Supplementation of the gestation diet with 0.5 ppm of mixed Se may improve piglet growth performance, increase Se concentrations in milk, and enhance Se status in the serum of sows, as well as in the serum and tissues of their offspring.

## INTRODUCTION

Selenium (Se) is a critical micronutrient in sow nutrition, and ensuring its optimal form and quantity is essential for maintaining sow health, productivity, and reproductive performance [1]. Se occurs in two primary chemical forms in nature: organic and inorganic. Organic Se, such as Se-enriched yeast and selenomethionine (SeMet), is typically considered superior to inorganic Se due to its higher bioavailability, lower environmental impact, and reduced toxicity [2]. In contrast, inorganic Se is passively absorbed and rapidly excreted, leading to less efficient Se retention in the body [3]. The form and concentration of Se in sow diets can significantly influence their health, reproductive efficiency, and the growth performance of their offspring.

The National Research Council [4] recommends a dietary Se level of 0.15 ppm for sows, while the U.S. Food and Drug Administration [5] permits up to 0.30 ppm in animal feed, irrespective of the Se source. Modern sows exhibit larger body sizes, produce more piglets per litter, and have higher milk production to support their larger litter [6]. Inadequate nutrient provision during gestation and lactation can adversely affect reproductive performance, longevity, and offspring growth [7]. Consequently, it is pertinent to consider whether the established essential nutrient levels should be reassessed to meet the increased demands of contemporary sows [8], particularly regarding Se sources and their higher inclusion levels (0.30 ppm vs 0.5 ppm) in gestating sows to enhance antioxidant status and reproductive performance.

Commercial Se-enriched yeast products predominantly contain selenium in their organic form, with approximately 55% to 75% as selenomethionine (SeMet) [9]. SeMet cannot be synthesized by animals and must be obtained through the diet [10]. Research indicates that Se yeast supplementation in pregnant sows offers significant benefits over traditional sodium selenite, including improved selenium status in sows and piglets [11], tissue Se deposition in newborn piglets [12], and Se content in colostrum and milk [13], all at supplementation levels of 0.15 ppm or 0.30 ppm. Maternal inorganic Se supplementation more effectively enhances the antioxidative status of sows and is more cost-effective than organic Se [14]. Thus, combining organic and inorganic Se sources may be a viable strategy to improve sow productivity and piglet growth while maintaining cost efficiency. However, most studies have examined the effects of Se sources at a supplementation level of 0.30 ppm, focusing mainly on late gestation and lactation. Data on the effects of combined Se sources at higher levels throughout the entire gestation period are limited.

This study examined the impact of high-level mixed selenium (Se) sources in the gestation diet on the performance of sow, serum Se concentrations in sows and piglets, selenium deposition in the tissues of newborn piglets. We hypothesized that supplementing gestating sows with a combination of organic and inorganic Se at elevated levels would enhance sow performance, improve piglet growth, and increase Se levels in both sows and piglets. Additionally, this study aimed to assess the feasibility of using combined Se sources in sow diets when Se levels exceed standard recommendations, in response to potential future increases in nutritional demands.

## MATERIALS AND METHODS

### Animals

All experimental procedures involving animals were conducted following the Animal Experimental Guidelines provided by the Seoul National University Institutional Animal Care and Use Committee (SNUIACUC; SNU-201112-3).

A total of 44 multiparous F1 sows (Yorkshire×Landrace), averaging 222.5 kg in body weight (BW), 18.61 mm in backfat (BF) thickness, and 4.2 in parity, were divided into one of three treatment groups in a completely randomized design with 14 or 15 replicates based on their BW, BF thickness, and parity. Artificial insemination service was performed two times a day and the interval was 12 hours when strong signs of the estrus were observed. Pregnancy was confirmed via ultrasound scanner (Donjin BLS, Gwangju-si, Gyeonggi-do, Korea) at day 35 of gestation. For the sow feeding program, all sows (parity, 3 to 6) were fed 2.4 kg/d with flat feeding.

### Experimental design and diet

The treatment diets were divided into three treatments, such as the control, the ISOS30 and the ISOS50 treatment. The control as a basal diet was designed to be a corn-soybean meal basal diet with no addition of Se premix. The ISOS30 treatment was supplemented with inorganic Se at 0.15 ppm and organic Se at 0.15 ppm. The ISOS50 treatment was supplemented with inorganic Se at 0.25 ppm and organic Se at 0.25 ppm. The selenium sources were sodium selenite (1,000 mg/kg, inorganic selenium) and selenium yeast (1,000 mg/kg, organic selenium, Sel-Plex; Alltech, Brookings, SD, USA). The selenium concentration in the gestation diet was shown: i) Control treatment, 0.05 ppm Se; ii) ISOS30 treatment, 0.34 ppm Se; iii) ISOS50 treatment, 0.61 ppm Se. All nutrients, including metabolizable energy, crude protein, lysine, methionine, calcium, and total phosphorus, were designed to meet the NRC [4] requirements. Table 1 showed the formulation of raw materials and chemical compositions in the gestating and lactating diets. After farrowing, all sows were fed the same lactation diet (same level of Se).

### Animal management

Experimental sows (parity, 3 to 6) were fed an experimental diet once daily at 8:00 a.m., with an initial amount of 2.4 kg per day during gestation. This amount was gradually reduced to 0.2 kg per day over the five days prior to farrowing. Post-farrowing, sows were transitioned to a lactation diet, increasing from 1 kg to 5 kg per day as lactation progressed, and were provided with an *ad libitum* diet before weaning.

The sows were individually housed in gestation stalls (2.20×0.64 m) with an automatic ventilation system that maintained a temperature of approximately 20°C. On day 110 of gestation, sows were transferred to farrowing crates (2.50×1.80 m) after being washed and disinfected, with special attention to their udders and vulvas. Delivery inducers were not used, but assistance was available for cases of dystocia. The lactation house was maintained at 28°C±2°C, with the piglet area under a heating lamp kept at 32°C±2°C. Air conditions and the temperature in the lactation house were controlled automatically by the ventilation system and air conditioning.

Piglets were cross-fostered within their treatment groups within the first 12 hours after birth to standardize suckling intensity among sows and equalize litter sizes, thus reducing the potential effects of initial litter size on growth. The umbilical cord and tail were cut, and castration was performed three days post-birth. Piglets were administered an injection of 150 ppm Fe-dextran (Gleptosil; Alstoe, York, UK). During the lactation period, creep feed was not provided to the piglets. Weaning occurred approximately 24±3 days after birth.

### Performance of the sow

During the gestation and lactation periods, sows’ BW was measured using a BI-2RB scale (BI series; CAS Co., Ltd., Seoul, Korea), and BF thickness was assessed at the P2 position with a Lean Meter (Renco Corp., Minneapolis, MN, USA). Measurements were taken on days 90, days 110 of gestation, 24 hours postpartum, and on day 21 of lactation. Daily feed wastage was recorded throughout lactation, and total feed intake over the 21-day lactation period was calculated to evaluate its physiological impact. The interval from weaning to the first estrus was recorded to determine the weaning-to-estrus interval (WEI).

The number of piglets born alive, stillborn, and mummified fetuses, as well as the total number born and their BWs, were recorded 24 hours after farrowing and on day 21 of lactation. Individual piglet weights and litter sizes were documented postpartum and on day 21 of lactation. The WEI of sows was recorded post-weaning as a key indicator of reproductive performance. Additionally, voluntary feed intake of sows was monitored throughout the lactation period.

### Reproductive performance and litter performance

Within 24 hours postpartum, the number of piglets born, including those alive, stillborn, and mummified, was recorded, along with their BWs. After farrowing, the number of piglets was recorded, and the BW of live piglets was measured by an electric scale (CAS CO. Ltd., Yangju, Korea). When measuring the BW of piglets, ear notching was practiced for the experiment. After ear notching, cross-fostering of the piglets within the same treatment was performed until 24 hrs postpartum to equalize litter size. The number and BW of piglets were measured again at day 21 of lactation to calculate litter weight, piglet weight, and weight gain.

### Blood and tissue samples

Blood was collected via jugular venipuncture from a randomly selected subset of pregnant sows (n = 8 per treatment) on days 90 and 110 of gestation, the day of parturition and 21 days after postpartum. The piglets (n = 20 per treatment) were bled via the anterior vena cava at 24 h postpartum and day 21 of lactation. Blood samples were collected in disposable culture tubes, and centrifuged at 1,957×g for 15 min (5810R; Eppendorf, Hamburg, Germany), the serum samples was collected and stored at −20°C until analysis.

Before the intake of colostrum, neonatal piglets were killed. Tissue samples, including liver, kidney, and muscle, were collected, frozen, and subsequently analyzed for selenium (Se) content.

### Milk composition

Colostrum was collected at 12 hours postpartum, and milk was collected on day 21 of lactation from the first and second teats of sows (n = 8 per treatment, using the same sows as for blood sampling). Before collection, sows were administered a 5 IU oxytocin injection (Komi oxytocin inj.; Komipharm International, Siheung, Korea) via the ear vein. The collected milk samples were stored frozen at −20°C until analysis. The nutritional composition of both colostrum and milk (day 21) was analyzed using a Milkoscan FT 120 (FOSS, Hillerød, Denmark) at the National Institute of Animal Science (Wanju, Korea).

### Se analytical method

Colostrum, milk, serum and tissues were analyzed according to the fluorometric method outlined by AOAC [15]. After the wet ashing of samples with nitric acid and perchloric acid at 160°C for approximately 2.5 to 3 hrs and the reduction with 6 mol/l hydrochloric acid, selenium was determined by 2,3-diamino-naphthalene fluorescence reaction.

### Statistical analysis

The experimental data were analyzed with the general linear model procedures of SAS [16] and performed using one-way analysis of variance procedure on the collected data. Individual sow, whole litter weight, average pig weight within the litter, serum and tissue samples were considered the experimental units. Contrasts were used to compare the control (0 Se) treatment to the combination of selenium source at levels of 0.30 or 0.50 ppm. Differences were considered significant at p<0.05 and highly significant at p<0.01, while a tendency was detected by p-value between p≥0.05 and p<0.10.

## RESULTS

### Performance of the sow

The effect of mixed Se source on sow performance was shown in Table 2. Body weight, BF thickness, lactation feed consumption, and the WEI of sows were not affected by mixed Se during the whole experimental period.

### Reproductive performance

Table 3 described the reproductive performance of sows in response to mixed levels of Se source. The mixed addition of Se did not influence on the number of piglets in total born, stillbirths, and born alive. There also were no significant differences in litter birth weight and piglet birth weight.

### Tissue selenium concentration in piglets

The effect of mixed Se source in the gestation diet on mixed Se concentration in piglet tissues was shown in Table 4. Before colostrum intake, newborn piglets whose sows were given either organic or inorganic selenium had significantly higher selenium levels in their kidneys than those whose sows were fed the control diet. The ISOS50 treatment, in particular, showed a notably greater increase (p = 0.01). ISOS treatments showed higher trends in the liver and muscle Se concentrations (p = 0.06; p = 0.09; respectively).

### Milk composition

Table 5 showed the effects of mixed Se supplementation on milk composition in lactating sows. The study revealed that the ISOS50 treatment had a significantly higher Se content in colostrum compared to the ISOS30 and control treatments (p<0.05). On day 21 of lactation, the ISOS treatments showed a numerical increase compared to the control group.

### Serum selenium concentration in gestating sows and piglets

The effects of mixed Se supplementation on serum Se level in sows and their progeny were shown in Table 6. The sow Se concentrations at days 90, and 110 of gestation and 24 hours postpartum were highest when sows were fed mixed addition of Se at a level of 0.50 ppm (p<0.05, respectively). Moreover, the treatment in a high level of mixed Se (0.50 ppm) had a higher piglet serum Se concentration than that in a low level (0.30 ppm) and control at 24 hours postpartum (p<0.05).

### Litter performance

On day 21 of lactation, piglets supplemented with a high level of mixed Se (0.50 ppm) had significantly greater BW and weight gain compared to those receiving a lower level of mixed Se (0.30 ppm), with differences showing statistical significance (p<0.05 for BW and p<0.03 for weight gain).

## DISCUSSION

Previous studies have evaluated individual factors such as selenium (Se) source or level on sow performance [12,17,18]. These studies reported that dietary Se sources provided to gestating sows did not significantly affect BW or BF thickness during gestation [19,20]. Additionally, variations in dietary Se levels (0 ppm, 0.3 ppm, 0.5 ppm) in gestating or lactating diet did not result in significant changes in BW or BF thickness before and after farrowing or during the 21-day lactation period [21,22]. Consistent with these findings, the current study further confirms that increasing Se supplementation up to 0.5 ppm in the gestating sow’s diet has no beneficial effects on BW, BF thickness, or their changes during gestation and lactation, regardless of whether a single Se source or a mixture was used.

Reduced feed intake during lactation could lead to abnormal weight loss and delayed reproductive cycles. Therefore, maximizing feed intake during lactation is recommended to support sustainable reproduction, as suggested by Eissen et al [23]. In this study, Se was provided at levels two to three times higher than the recommended amount in NRC [4], which did not affect feed consumption during lactation. Falk et al [24] found that sows fed Se yeast during late gestation and lactation had higher feed intake compared to those fed inorganic Se. Similarly, Kim et al [25] reported numerically higher average daily feed intake (ADFI) in lactating sows treated with Se yeast due to its better flavor of yeast. In contrast, the current study observed that long-term intake of mixed Se source in the gestating diet did not affect ADFI during lactation. This observation might be related to the use of the same commercial feed provided to the experimental sows during the lactation period

Various sources or levels of Se in dietary supplementation have been tested, but they did not affect sow reproductive performance [21,26]. Mahan and Kim [12] observed that gilt reproductive performance from 60 days before breeding to weaning was unaffected by both the dietary Se level (0.1 ppm or 0.3 ppm) and the Se source (selenite or selenium yeast). Mahan [13] also found that the Se source provided during late pregnancy did not impact the number of pigs per litter, stillborn, or piglet birth weight. Similarly, Svoboda et al [27] reported no significant difference in sow reproductive performance was found when sows were given either inorganic Se or Se yeast (0.3 ppm for gestation and 0.38 ppm for lactation). This experiment also demonstrated similar results, indicating no improvement in sow reproductive performance with the Se mixture at either of the two levels. However, Mahan and Peter [20] observed the number of stillborn was lower in sows that received Se yeast in their diets over four consecutive parities (1 to 4 parities). Likewise, Mou et al [18] reported that supplementing the sow’s diet with 0.30 ppm of organic Se can reduce the birth interval of piglets, shortening the total farrowing duration. Chen et al [17] also found that gestating sows fed organic Se (Se yeast) at 0.30 ppm had better litter birth weights than those fed inorganic Se. In the current study, mixed Se treatments and the control did not find detectable effects on the number of stillborn and litter birth weights. The differences in these results may be attributed to the study being confined to a single reproductive cycle and the use of multiparous sows with varying selenium (Se) status before the study started, as well as other factors such as Se source, Se level, sow parity, reproductive stage, and feeding program.

Se concentrations vary significantly among newborn piglet tissues, with levels decreasing in the following order: kidney, liver, and muscle [28]. Mahan and Kim [12] stated that organic Se sources are more effectively transferred through the placenta, resulting in higher Se levels in the loin and liver of newborn piglets. Several studies have shown that increasing Se supplementation from 0.1 to 0.3 mg/kg, whether from an inorganic or organic source, generally increases Se concentrations in both neonatal and weaning piglet loin [20, 29–31]. The present study found that Se concentrations in organ tissues were significantly higher in the ISOS50 group compared to the ISOS30 and control groups. Supplementation with 0.5 ppm of mixed Se sources notably increased Se levels in newborn piglets, indicating effective maternal transfer. However, accurately quantifying the amount of Se transferred to the fetus through the placenta remains challenging due to the sows’ Se metabolism needs and requires further research. Furthermore, Se deposition in muscle tissues is associated with piglet performance [32]. Se reserves in muscle, particularly as SeMet, may enhance the piglet’s response to stress as well as its health and growth [33]. Therefore, the higher Se concentration in the muscles of piglets from the ISOS50 group may partially contribute to the observed improvements in BW and average daily gain by day 21 of lactation.

In general, the nutritional composition of sow feed aligns well with the composition of colostrum and milk in gestating sows [34]. The concentration of Se in colostrum and milk is primarily influenced by the form of Se provided to the sows [35]. Specifically, as the level of organic Se in the sow’s diet increases, the Se content in both colostrum [12,13, 20,29,30,36] and milk [13,20,27,29,30] was increased linearly compared to inorganic Se source with high level. Our data showed that ISOS treatments resulted in higher Se content in colostrum compared to the control group, with the highest Se levels observed in the ISOS50 treatment as organic Se levels increased. Surai et al [33] stated that organic Se dietary supplementation resulted in higher Se concentrations in milk, which was attributed to the non-specific incorporation of SeMet into milk proteins. This study also aligns with Mahan [13], who reported that organic Se with higher Se levels (0.15 ppm or 0.30 ppm) is more efficiently stored during pregnancy and readily transferred to colostrum. However, the Se content in milk at day 21 of lactation did not differ significantly among treatment groups, and Selenium (Se) concentrations in the ISOS group were only numerically higher than those in the control group. This may be due to the depletion or transfer of Se stored in sows during pregnancy, which did not result in significant differences, or it may be attributed to the consistent selenium levels in the lactation feed.

In the current study, serum Se concentrations of sows and piglets were increased by high levels of mixed Se at measured times, except at 21 days of lactation. Duntas and Benvenga [37] reported that organic Se is more efficient than inorganic Se at elevating Se level in the blood and has higher deposition in the animal. The serum Se concentration of sows demonstrated a positive correlation with increasing levels of Se supplementation [13]. Mahan and Peter [20] also reported that as organic Se or inorganic Se levels increased (0 ppm, 0.15 ppm, 0.30 ppm), there was a further increase in sow blood and tissue Se concentrations. This indicated that the changes in Se concentration in sows’ serum are solely determined by the level of Se supplementation in the sow diet. However, in piglets, the increase in serum Se concentration after birth was determined by the colostrum intake and stored Se in tissues. As this experiment revealed, the ISOS50 treatment group, which received a higher level of mixed Se, resulted in increased Se transfer to the newborn piglets, allowing for greater Se storage in piglet tissue (Table 4). As previously discussed, it can be predicted that serum Se concentrations in sows and piglets were positively influenced by the inclusion of organic Se and high levels of Se (0.30 ppm vs 0.50 ppm) in this study. In contrast, Kim et al [25] reported that the combination of Se (0.25 ppm organic + 0.25 ppm inorganic) had similar serum Se concentrations in sows and piglets compared with mixed Se source (0.15 ppm organic + 0.15 ppm inorganic) in lactating sows. These differences are likely related to the gestation period with Se supplementation, which had a sensitively long-term effect on the change of serum Se concentration in both sows and piglets.

Several studies have indicated that maternal supplementation with Se yeast has minimal impact on the growth performance of offspring compared to inorganic Se sources [20,27,29,38]. However, research by Zhan et al [39] and Hu et al [36] demonstrated that maternal intake of organic Se enhanced Se concentration and antioxidant status in gestating sows, which supported maternal health and improved piglet growth, with these benefits being subsequently transferred to the offspring. A recent study by Jin et al [21] also reported that piglet BW and weight gain at 21 days of lactation were positively affected by the Se source or Se level, particularly with high levels of organic Se (0.50 ppm) in gestating sows. In the current study, piglet BW and weight gain were significantly increased when an increased Se mixture was supplied. The improvement in piglet BW and weight gain in the ISOS groups can be attributed to three factors. First, a higher tissue Se concentration was observed in newborn piglets due to greater transfer of Se from the sow to the conceptus at birth. Second, the transfer of Se through colostrum resulted in increased Se delivery to the offspring. Quesnel et al [40] reported that the minimum colostrum intake for newborn piglets is 250 grams. Based on Se content in the colostrum per piglet, the Se intake for each treatment group can be calculated as follows: Control group, 0.032 mg; ISOS30 group, 0.042 mg; ISOS50 group, 0.065 mg. Third, milk production may be increased with higher levels of mixed Se. According to the formula provided by Wang et al [41], the estimated total milk production for each treatment group (not shown in the data) is as follows: Control group, 178.19 kg; ISOS30 group, 183.92 kg; ISOS50 group, 189.64 kg. The Se stored in newborn piglet tissues and the higher Se concentration in colostrum effectively improved the Se status in piglets, leading to increased BW and daily weight gain. The increased milk yield in sows indicated that piglets in the high-level mixed Se supplementation group consumed more milk during lactation, enhancing the growth of piglets.

## CONCLUSION

In conclusion, high levels of mixed organic and inorganic Se supplementation significantly increased Se concentrations in the serum of sows and piglets, enhanced Se content in colostrum and milk, and improved Se accumulation in piglet tissues, thereby improving piglet BW and weight gain. However, there were no significant effects on sow performance during gestation and lactation. Based on these results, we recommend supplementing the gestating diet with 0.5 ppm of mixed Se to achieve optimal piglet BW and weight gain. Future research should examine the long-term effects of different levels and ratios of mixed selenium sources on sows and their offspring. Additionally, it is important to assess the individual contributions of organic and inorganic selenium sources when using mixed selenium sources in sow diets.

## Figures and Tables

**Table 1:** Formulations and chemical compositions of the experimental diets

Items	Gestating diet^1)^	Lactating diet
Ingredients (%)		
Maize	77.96	65.34
SBM, 48% CP	12.00	27.24
Wheat bran	5.00	2.00
Tallow	1.35	2.41
Lysine HCl (50%)	0.34	-
Methionine (99%)	0.02	-
Dicalcium phosphate	1.28	1.26
Limestone	1.35	1.13
Vitamin. Mix^2)^	0.10	0.10
Mineral. Mix^3)^	0.10	0.10
Choline chloride-50%	0.10	0.10
Salt	0.40	0.30
Sum	100.00	100.00
Chemical composition^4)^		
ME (kcal/kg)	3,265.00	3,300.00
CP (%)	12.00	17.21
SID Lysine (%)	0.74	0.94
SID Methionine (%)	0.23	0.29
Calcium (%)	0.75	0.75
Total phosphrous (%)	0.60	0.60

CP, crude protein; ME, metabolizable energy; SID, standardized ileal digestible.

1) Sodium selenite and Se yeast, each providing a total Se concentration of 1,000 mg/kg, were supplemented to the basal diet to achieve the specified treatment levels. For the ISOS30 group, the diet was supplemented with 0.15 ppm inorganic Se and 0.15 ppm organic Se. For the ISOS50 group, the diet was supplemented with 0.25 ppm inorganic Se and 0.25 ppm organic Se.

2) Provided the following per kilogram of gestation diet: vitamin A, 8,000 IU; vitamin D_3_, 1,600 IU; vitamin E, 32 IU; d-biotin, 64 g; riboflavin, 3.2 mg; calcium pantothenic acid, 8 mg; niacin, 16 mg; vitamin B_12_, 12 g; vitamin K, 2.4 mg. Provided per kg of lactation diet: vitamin A, 10,000 IU; vitamin D_3_, 1,900 IU; vitamin E, 80 IU; vitamin K_3_, 3.25 mg; thiamine (vitamin B_1_), 2.00 mg; riboflavin (vitamin B_2_), 7.0 mg; pantothenic acid (vitamin B_5_), 27.5 mg; niacin (vitamin B_3_), 36 mg; pyridoxine (vitamin B_6_), 3.75 mg; d-biotin, 0.35 mg; folic acid, 2.25 mg; vitamin B_12_, 0.03 mg.

3) Provided the following per kilogram of gestation diet: Se, 0 mg; I, 0.3 mg; Mn, 24.8 mg; CuSO4, 54.1 mg; Fe, 127.3 mg; Zn, 84.7 mg; Co, 0.3 mg. Provided per kg of lactation diet: Se, 0.15 mg; I, 0.3 mg; Mn, 37 mg; Cu, 11 mg; Fe, 150 mg; Zn, 85 mg; Co, 2 mg.

4) Calculated value.

**Table 2:** Influences of mixed selenium supplementation in gestating diet on BW, BF thickness, and their changes, WEI, and lactating feed consumption of sows^1)^

Items	Treatment^2)^			SEM	p-value

	Control	ISOS30	ISOS50		
No. of sows farrowed		15	14	15	
Body weight (kg)					
At mating	222.83	221.83	222.63	5.96	0.99
35 d	226.67	225.33	230.50	4.68	0.92
110 d	257.20	262.57	258.13	4.88	0.92
Change (0 to 110 d)	34.37	40.73	35.30	4.41	0.86
24 h postpartum	238.41	240.27	239.47	3.31	0.98
21 d of lactation	230.89	236.10	232.00	3.61	0.86
Change (0 to 21 d)	−7.52	−4.17	−7.47	3.29	0.92
Backfat thickness (mm)					
At mating	18.83	18.32	18.68	1.31	0.99
35 d	18.87	18.32	18.75	1.33	0.98
110 d	22.32	22.31	22.94	1.05	0.97
Backfat gain (0 to 110 d)	3.50	3.97	4.27	0.56	0.88
24 h postpartum	20.19	21.67	20.71	1.13	0.89
21 d of lactation	18.97	19.71	20.07	1.06	0.93
Backfat change (0 to 21 d)	−1.22	−1.96	−0.63	0.34	0.31
Lactation feed intake (kg/d)	5.82	5.62	5.73	0.46	0.40
WEI (d)	4.50	4.50	3.75	0.25	0.41

SEM, standard error of the mean; WEI, weaning to estrus interval.

1) Changes in body weight and back fat of sows during gestation and lactation periods, and their feed intake during lactation.

2) Treatment: Control, corn-soybean meal-based diet with no addition of selenium premix; ISOS treatment group, the ratio of organic selenium to inorganic selenium was 1:1.

**Table 3:** Influences of mixed selenium supplementation on reproductive performance of sow^1)^

Items	Treatment^2)^			SEM	p-value

	Control	ISOS30	ISOS50		
No. of sows		15	14	15	
No. of piglets					
Total born	13.25	13.25	13.25	0.179	1.00
Stillbirth	0.50	0.50	0.50	0.151	1.00
Mummy	0.50	0.50	0.75	0.231	0.67
Born alive	12.25	12.25	12.00	0.112	0.62
Litter weight					
Total litter weight (kg)	19.32	18.97	19.05	0.448	0.96
Alive litter weight (kg)	18.43	18.01	18.15	1.339	0.95
Piglet weight					
Piglet birth weight (kg)	1.51	1.48	1.51	0.038	0.93

SEM, standard error of the mean.

1) Reproductive performance data of Sows after farrowing, such as number of piglets, litter weight, and birth weight of newborn piglets.

2) Treatment: Control: corn-soybean meal-based diet with no addition of selenium premix. In the ISOS treatment group, the ratio of organic selenium to inorganic selenium was 1:1.

**Table 4:** Influences of mixed selenium supplementation in gestation diet on tissue Se concentration in newborn piglets

Tissue selenium (ppm)	Treatment^1)^			SEM	p-value

	Control	ISOS30	ISOS50		
Liver	0.317^b^	0.409^ab^	0.490^a^	0.032	0.06
Kidney	0.739^c^	0.813^b^	0.837^a^	0.022	0.01
Muscle	0.152^b^	0.166^ab^	0.178^a^	0.005	0.09

SEM, standard error of the mean.

1) Treatment: Control, corn-soybean meal-based diet with no addition of selenium premix; ISOS treatment group, the ratio of organic selenium to inorganic selenium was 1:1.

a–c Means with different superscripts in the same row significantly differ (p<0.05).

**Table 5:** Influences of mixed selenium supplementation in gestation diet on milk composition in lactating sows

Items	Treatment^1)^			SEM	p-value

	Control	ISOS30	ISOS50		
Selenium (ppm)					
Colostrum	0.126^c^	0.169^b^	0.260^a^	0.02	0.05
Milk (21 d)	0.098	0.104	0.114	0.04	0.69
Casein (%)					
Colostrum	7.74	7.77	7.72	0.16	0.99
Milk (21 d)	3.97	3.96	4.02	0.08	0.95
Fat (%)					
Colostrum	5.64	5.65	5.64	0.33	1.00
Milk (21 d)	7.05	6.85	7.53	0.21	0.46
Protein (%)					
Colostrum	11.91	11.86	11.88	0.17	0.99
Milk (21 d)	4.84	4.77	4.89	0.12	0.94
Lactose (%)					
Colostrum	3.50	3.50	3.51	0.05	1.00
Milk (21 d)	5.62	5.78	5.60	0.06	0.51
Total solid (%)					
Colostrum	23.38	23.40	23.38	0.37	1.00
Milk (21 d)	19.11	18.49	19.56	0.33	0.48
Solid not fat (%)					
Colostrum	17.08	17.06	17.13	0.36	1.00
Milk (21 d)	11.13	11.22	11.20	0.10	0.95

SEM, standard error of the mean.

1) Treatment: Control, corn-soybean meal-based diet with no addition of selenium premix; ISOS treatment group, the ratio of organic selenium to inorganic selenium was 1:1.

a–c Means with different superscripts in the same row significantly differ (p<0.05).

**Table 6:** Influences of mixed selenium supplementation in gestation diet on serum Se concentration of sows and their piglets

Items	Treatment^1)^			SEM	p-value

	Control	ISOS30	ISOS50		
Sow serum selenium (ppm)					
At mating	--------------------------------------- 0.249 ----------------------------------------				
90 d	0.125^c^	0.134^b^	0.176^a^	0.018	0.02
110 d	0.204^c^	0.223^b^	0.265^a^	0.010	0.02
24 h postpartum	0.172^b^	0.211^ab^	0.239^a^	0.011	0.01
21 d of lactation	0.217	0.257	0.248	0.009	0.21
Piglets serum selenium (ppm)					
24 hours postpartum	0.126^c^	0.169^b^	0.260^a^	0.006	0.05
Day 21 of lactation	0.098	0.104	0.114	0.005	0.26

SEM, standard error of the mean.

1) Treatment: Control: corn-soybean meal-based diet with no addition of selenium premix. In the ISOS treatment group, the ratio of organic selenium to inorganic selenium was 1:1.

a–c Means with different superscripts in the same row significantly differ (p<0.05).

**Table 7:** Influences of mixed selenium supplementation in gestation diet on litter performance

Items	Treatment^1)^			SEM	p-value

	Control	ISOS30	ISOS50		
No. of piglets					
After cross-foster^1)^	12.00	12.00	12.25	0.083	0.40
21 days of lactation	11.00	11.00	11.00	0.246	0.98
Litter weight (kg)					
After cross-foster	18.08	18.05	18.21	1.339	0.30
21 days of lactation	61.17	62.48	63.80	4.585	0.48
Litter weight gain (0 to 21 d)	43.09	44.43	45.59	1.631	0.81
Piglet body weight (kg)					
After cross-foster^2)^	1.51	1.50	1.49	0.035	0.98
21 days of lactation	5.56^b^	5.68^ab^	5.80^a^	0.037	0.05
Piglet weight gain (0 to 21 d)	4.05^b^	4.18^ab^	4.31^a^	0.046	0.03

SEM, standard error of the mean.

1) Treatment: Control, corn-soybean meal-based diet with no addition of selenium premix; ISOS treatment group, the ratio of organic selenium to inorganic selenium was 1:1.

2) After cross-fostering day within 12 hours postpartum.

a,b Means within each row with different superscripts differ (p<0.05).
